# Health Disparities in Kidney Failure Among Patients With Autosomal Dominant Polycystic Kidney Disease: A Cross-Sectional Study

**DOI:** 10.1016/j.xkme.2022.100577

**Published:** 2022-12-05

**Authors:** Teresa N. Harrison, Qiaoling Chen, Min Young Lee, Mercedes A. Munis, Kerresa Morrissette, Shirin Sundar, Kristin Pareja, Ali Nourbakhsh, Yu-Hsiang Shu, Cynthia J. Willey, John J. Sim

**Affiliations:** a Department of Research & Evaluation, Kaiser Permanente Southern California, Pasadena, California; b Division of Nephrology and Hypertension, Kaiser Permanente Los Angeles Medical Center, Los Angeles, California; c Otsuka Pharmaceutical Development & Commercialization, Inc, Princeton, New Jersey; d Department of Biostatistics and Programming, Inari Medical, Irvine, California; e College of Pharmacy, University of Rhode Island, Kingston, Rhode Island; f Department of Clinical Science, Kaiser Permanente Bernard J. Tyson School of Medicine, Pasadena, California

## Abstract

**Rationale & Objective:**

Understanding potential differences in patterns of kidney failure among patients with autosomal dominant polycystic kidney disease (ADPKD) may provide insights into improving disease management. We sought to characterize patients with ADPKD and kidney failure across different race/ethnicities.

**Study Design:**

Cross-sectional study.

**Setting & Participants:**

Kaiser Permanente Southern California members diagnosed with ADPKD between January1, 2002, and December 31, 2018.

**Exposure:**

ADPKD.

**Outcome:**

Kidney failure, dialysis, or receipt of kidney transplant.

**Analytical Approach:**

Differences in characteristics by race/ethnicity were assessed using analysis of variance F test and χ^2^ test. To compare the range and distribution of the average age at onset of kidney failure by race/ethnicity and sex, we used box plots and confidence intervals. Multivariable logistic regression was used to estimate OR for kidney transplant.

**Results:**

Among 3,677 ADPKD patients, 1,027 (27.3%) had kidney failure. The kidney failure cohort was comprised of Black (n=138; 30.7%), White (n=496; 30.6%), Hispanic (n=306; 24.7%), and Asian (n=87; 23.6%) patients. Hispanic patients had the youngest mean age of kidney failure onset (50 years) compared to Black (56 years) and White (57 years) patients. Black (44.2%; OR, 0.72) and Hispanic (49.7%; OR, 0.65) patients had lower rates of kidney transplantation compared to White (53.8%) patients. Preemptive kidney transplantations occurred in 15.0% of patients.

**Limitations:**

Retrospective study design and possible misclassification of ADPKD cases. Kidney function calculations were based on equations incorporating race, potentially overestimating kidney function in African Americans. The study was conducted within a single, integrated health care system in 1 geographic region and may not be generalizable to all ADPKD patients.

**Conclusions:**

Among a large diverse ADPKD population, we observed racial/ethnic differences in rates of kidney failure, age of kidney failure onset, and rates of kidney transplantation. Our real-world ADPKD cohort provides insight into racial/ethnic variation in clinical features of disease and potential disparities in care, which may affect ADPKD outcomes.

## Graphical abstract

**Figure:**
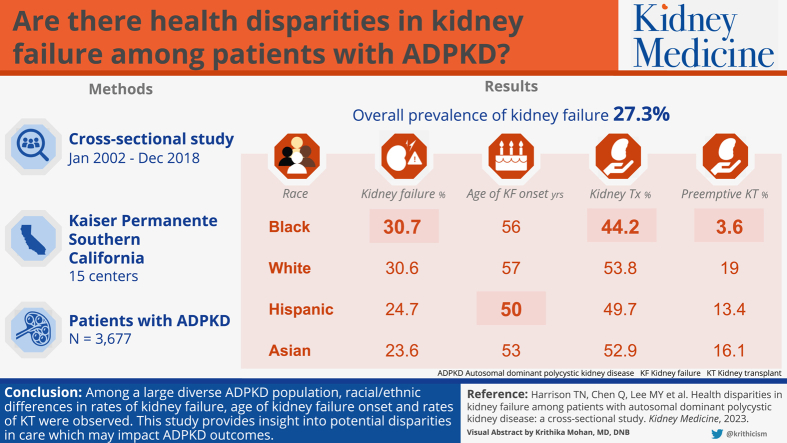

**Plain-Language Summary**

Among a large racially and ethnically diverse population of 3,677 American patients with autosomal dominant polycystic kidney disease (ADPKD) from an integrated health care system, 27% had kidney failure. We observed racial/ethnic differences in rates of kidney failure, age of kidney failure onset, and rates of kidney transplantation. A higher percentage of Black and non-Hispanic White patients had kidney failure compared to Hispanic and Asian patients. Asian and Hispanic patients had kidney failure at an earlier age compared to White and Black patients. Black and Hispanic patients were less likely to have kidney transplants compared to non-Hispanic White and Asian patients. Our real-world ADPKD cohort provides insight into racial/ethnic variation in clinical features of disease and potential disparities in care, which may affect ADPKD outcomes.

Autosomal dominant polycystic kidney disease (ADPKD) is the most common genetic disorder affecting the kidney with a prevalence of around 1 in 500-2000 people.^1^, ^2^, ^3^ The natural progression of the disease is characterized by pathogenic genetic mutations that lead to fluid-filled cyst development, causing irreversible destruction of kidney parenchyma and function.^4^ This can ultimately lead to the need for kidney replacement therapy, making it the fourth leading cause of kidney failure.^5^ Approximately 50% of patients with ADPKD progress to kidney failure by age 59 years in males and 64 years in females.^6^ ADPKD is also a systemic disorder, which may contribute to its increased morbidity and negative outcomes, especially in the kidney failure population. Because of the progressive nature of the disease, there is strong interest in better understanding the natural course of ADPKD. Better insights into ADPKD could lead to earlier detection in life before the onset of kidney failure and could help implement management strategies to delay progression. Current management strategies include blood pressure control, exercise, low-salt diet, and hydration. Although there is no cure for ADPKD, tolvaptan, a selective vasopressin V_2_ receptor blocker, has been Food and Drug Administration approved as the first treatment to slow kidney function decline in adults at risk of rapidly progressing ADPKD.^7^^,^^8^ In addition, earlier identification and follow-up with a nephrologist may lead to more optimal management of patients with ADPKD, including earlier listing for kidney transplant.^9^

Identified risk factors for rapid progression of ADPKD to kidney failure include early age onset of gross hematuria and hypertension, high body mass index (BMI), proteinuria, large total kidney volume, and certain genetic mutations.^10^, ^11^, ^12^, ^13^ However, the role of race and ethnicity on disease progression has not been elucidated. The risk calculator for ADPKD progression developed from the Consortium for Radiologic Imaging Studies of Polycystic Kidney Disease cohort and Mayo database used height-adjusted total kidney volume, age, sex, and creatinine but only accounted for race to calculate the estimated glomerular filtration rate (eGFR). Hence, race was not considered in the overall risk of progression.^14^ Additionally, the Predicting Renal Outcome in Polycystic Kidney Disease score is a prognostic algorithm developed to determine the risk of progression of kidney failure in ADPKD based on genotype and clinical factors. Overall, the Mayo imaging classification and the Predicting Renal Outcome in Polycystic Kidney Disease prognostic score were primarily derived from White populations, and thus, the impact of different race/ethnicities on ADPKD outcomes could not be determined.^15^^,^^16^

An improved understanding of racial/ethnic differences in incidence and progression of ADPKD would provide insights into better managing this population. Race/ethnicity is associated with known risk factors for ADPKD progression, such as high BMI and hypertension.^17^ Thus, race encompasses variables related to genetic, cultural, or health care differences that affect progression. Whether penetrance of ADPKD genes varies by race/ethnicity is yet to be determined. Although a study found no difference in the age of onset of kidney failure between Black and White patients with ADPKD,^18^ subsequent studies found that kidney failure occurred at a younger age in non-Hispanic Black patients compared to non-Hispanic White patients.^19^^,^^20^ Murphy et al^19^ observed lower overall incidence of kidney failure from ADPKD in non-Hispanic Black patients. These limited findings suggest a need to evaluate the impact of race on ADPKD progression to kidney failure.

Using a large racially/ethnically diverse ADPKD population from a real-world environment, we sought to characterize patients with ADPKD and kidney failure. Furthermore, we evaluated kidney failure rates, age of kidney failure onset, and kidney transplant rates by different race/ethnicities.

## Methods

### Study Setting and Population

This cross-sectional study was conducted among members of Kaiser Permanente Southern California (KPSC), a large integrated United States (US) health care system which provides care to nearly 4.8 million members at 15 medical centers and over 230 medical offices. The member population at KPSC is socioeconomically diverse and broadly representative of the racial/ethnic groups in Southern California.^21^ All aspects of medical care are captured in clinical and administrative databases using comprehensive electronic health records.

**Figure 1:**
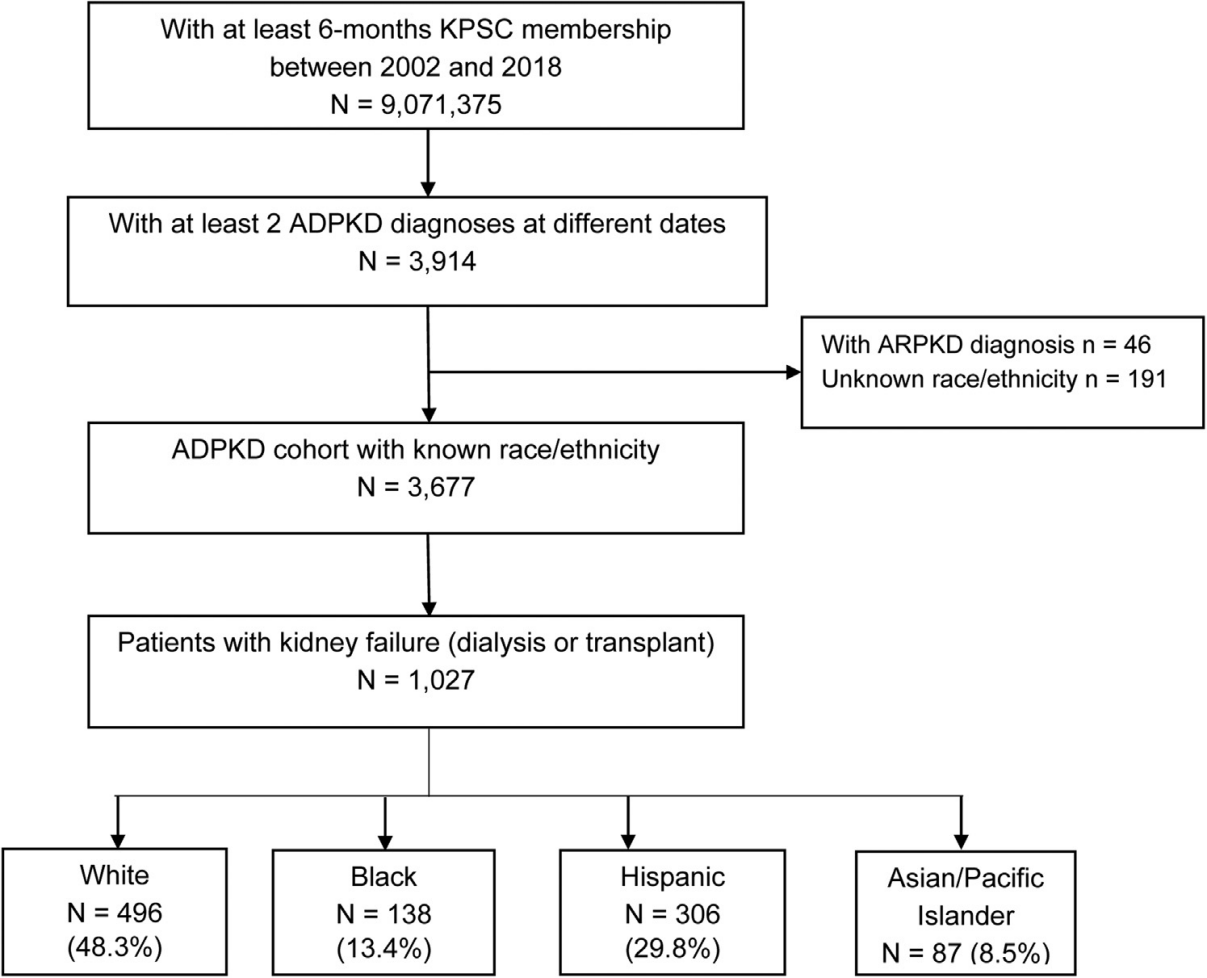
Cohort assembly of patients with ADPKD and kidney failure. A total of 3,677 prevalent patients with ADPKD were identified at KPSC in the period 2002-2018. Overall, 27.3% (n=1,027) of patients with ADPKD had kidney failure. Abbreviations: ADPKD, autosomal dominant polycystic kidney disease; KPSC, Kaiser Permanente Southern California.

Details of the KPSC population with ADPKD have been described previously.^22^ In brief, we identified all patients who had an ADPKD diagnosis between January 1, 2002 and December 31, 2018 with ≥ 2 ADPKD inpatient and/or outpatient diagnosis codes on separate encounter dates identified by *International Classification of Diseases, Ninth and Tenth Revision* (ICD-9: 753.12, 753.13; ICD-10: Q61.2, Q61.3) (Fig 1). The second encounter date was defined as the index date. Patients were required to have at least 6 months of KPSC membership before the beginning of the study period, January 1, 2002, or the index ADPKD diagnosis date, whichever was later. Patients who had a diagnosis of autosomal recessive polycystic kidney disease or who did not have at least 6 months of continuous KPSC membership were excluded from the study. In addition, patients with race/ethnicity identified as unknown or other were excluded.

The analytic sample was comprised of a subgroup of prevalent patients with ADPKD and kidney failure (n=1,027). Kidney failure was defined as kidney replacement therapy with hemodialysis, peritoneal dialysis, or kidney transplant. The date of kidney failure onset was the first date of hemodialysis, peritoneal dialysis, or kidney transplant. The initiation date of dialysis or receipt of kidney transplant was obtained from the KPSC Renal Business Group, which maintains a registry and follows all kidney failure patients within KPSC. Furthermore, we evaluated the proportion of kidney transplant patients, including those who had kidney transplant at kidney failure onset (preemptive) before treatment with any dialysis.

### Demographics and Clinical Characteristics of the ADPKD Kidney Failure Population

Patient demographics, clinical characteristics, and medications were assessed in the 1 year before the kidney failure onset date. Age, sex, race/ethnicity, BMI, antihypertensive agents (angiotensin-converting enzyme inhibitors/angiotensin receptor blockers, beta blockers, calcium channel blockers) were obtained from the electronic health record. Race and ethnicity were categorized into mutually exclusive groups: Hispanic (regardless of race; > 95% of KPSC patients identify as White), non-Hispanic Asian or Pacific Islander (Asian), Black, and White. Education and income were based on 2015-2019 American Community Survey data at the census tract level.

Baseline laboratory values (eg, serum creatinine, hemoglobin, lipid panels, and urine studies) were collected closest to and within 1 year before the kidney failure onset date. Kidney function was expressed as eGFR calculated from serum creatinine levels using the Chronic Kidney Disease Epidemiology Collaboration equation.^23^ Proteinuria was defined as positive for protein in urinalysis (qualitative tests), urine protein quantitation ≥200, urinary protein-creatinine ratio >0.2, or urinary albumin-creatinine ratio >30. Any history of comorbid conditions (hyperlipidemia, hypertension, diabetes, abdominal pain, urologic diseases, and the Elixhauser Comorbidity Index) were extracted from the electronic health record. Diabetes was identified using 1 or more of the following: diagnosis codes, antidiabetic medication use, and/or abnormal hemoglobin A_1C_ laboratory tests. The Elixhauser Comorbidity Index is a method of categorizing 31 comorbid conditions using diagnosis codes with each comorbid condition being unweighted and assigned a value of “1.”^24^

### Statistical Analyses

Descriptive statistics stratified by race/ethnicity were used to report age at identification of ADPKD among prevalent patients, and sociodemographic characteristics, clinical values, comorbid conditions, and antihypertensive medications at kidney failure onset. Differences were assessed using analysis of variance F test for continuous variables and χ^2^ test for categorical variables. Patients with kidney failure before January 1, 2002 were excluded from the baseline laboratory results because dialysis or a kidney transplant may have affected their results following treatment. Baseline laboratory results for patients identified with ADPKD before kidney failure were calculated separately. Patients were stratified by race/ethnicity. The proportion of patients with kidney failure and the average age at onset of kidney failure were calculated. To compare the range and distribution of the average age at onset of kidney failure by race/ethnicity and sex, we used box plots and confidence intervals.

Among patients with ADPKD and kidney failure, we performed a multivariable logistic regression model to estimate odds ratios (ORs) for kidney transplant using preselected variables that included race/ethnicity, age, sex, BMI, comorbid condition score, census-level education, and income data. All analyses were performed using SAS version 9.4.

The KPSC Institutional Review Board reviewed and approved the protocol of this study (#11823). A waiver of informed consent was obtained because of the retrospective nature of this study.

## Results

A total of 3,677 prevalent patients with ADPKD were identified during the study period (Fig 1). The mean age at the time of ADPKD identification was 52 (years) for White, 53 for Black, 43 for Hispanic, and 49 for Asian patients (Table 1). Overall, 27.3% (n=1,027) of patients with ADPKD had kidney failure, either at the time of diagnosis or later during the study period. A higher percentage of Black (n=138; 30.7%) and White patients (n=496; 30.6%) had kidney failure than Hispanic (n=306; 24.7%) and Asian patients (n=87; 23.6%) (Table 1).

**Table 1:** Baseline Characteristics of Patients with ADPKD and Kidney Failure

Characteristics^a^	Total	White	Black	Hispanic	Asian/Pacific Islander	*P*^b^
**ADPKD sample size**	3,677 (100.0%)	1,621 (44.1%)	450 (12.2%)	1,237 (33.6%)	369 (10.0%)	
**Mean duration of KPSC membership (years)**	14.0	19.3	19.5	13.0	12.6	
**Age at ADPKD identification, mean (SD)**	48.7 (18.2)	52.1 (17.8)	53.1 (18.4)	42.8 (17.6)	49.2 (18.2)	<0.01
**Kidney failure, n (%)**	1027 (27.3%)	496 (30.6%)	138 (30.7%)	306 (24.7%)	87 (23.6%)	<0.01
**n (%)**	1027 (100.0%)	496 (48.3%)	138 (13.4%)	306 (29.8%)	87 (8.5%)	
**Age at kidney failure onset, y**						
Mean (SD)	54.6 (13.5)	57.3 (12.9)	56.4 (14.6)	50.0 (12.9)	53.3 (12.5)	<0.01
<45, n (%)	220 (21.4%)	72 (14.5%)	31 (22.5%)	99 (32.4%)	18 (20.7%)	<0.01
45-54, n (%)	356 (34.7%)	173 (34.9%)	37 (26.8%)	115 (37.6%)	31 (35.6%)	
55-64, n (%)	230 (22.4%)	119 (24.0%)	33 (23.9%)	54 (17.6%)	24 (27.6%)	
65+, n (%)	221 (21.5%)	132 (26.6%)	37 (26.8%)	38 (12.4%)	14 (16.1%)	
**Sex, n (%)**						0.57
Male	558 (54.3%)	269 (54.2%)	71 (51.4%)	165 (53.9%)	53 (60.9%)	
**BMI**						<0.01
Mean (SD)	28.2 (6.3)	27.9 (5.8)	27.2 (5.9)	29.6 (7.1)	26.2 (5.1)	
**College degree**^c^^,^^e^**, n (%)**						<0.01
<50%	326 (31.7%)	90 (18.1%)	51 (37%)	169 (55.2%)	16 (18.4%)	
50-75%	406 (39.5%)	205 (41.3%)	60 (43.5%)	100 (32.7%)	41 (47.1%)	
>75%	267 (26%)	185 (37.3%)	22 (15.9%)	34 (11.1%)	26 (29.9%)	
Unknown	28 (2.7%)	16 (3.2%)	5 (3.6%)	3 (1%)	4 (4.6%)	
**Median household income**^c^^,^^e^**, n (%)**						<0.01
≤$45,000	107 (10.4%)	35 (7.1%)	23 (16.7%)	43 (14.1%)	6 (6.9%)	
$45,001-$80,000	366 (35.6%)	145 (29.2%)	50 (36.2%)	147 (48%)	24 (27.6%)	
>$80,000	486 (47.3%)	278 (56%)	56 (40.6%)	103 (33.7%)	49 (56.3%)	
Unknown	68 (6.6%)	38 (7.7%)	9 (6.5%)	13 (4.2%)	8 (9.2%)	
**Elixhauser comorbidity index**						
Mean (SD)	4.7 (2.8)	4.9 (2.92)	5.3 (2.77)	4.4 (2.58)	4.6 (2.91)	0.01
<3, n (%)	194 (18.9%)	98 (19.8%)	13 (9.4%)	62 (20.3%)	21 (24.1%)	0.05
3-4, n (%)	315 (30.7%)	140 (28.2%)	47 (34.1%)	106 (34.6%)	22 (25.3%)	
5-6, n (%)	289 (28.1%)	137 (27.6%)	45 (32.6%)	82 (26.8%)	25 (28.7%)	
7+, n (%)	229 (22.3%)	121 (24.4%)	33 (23.9%)	56 (18.3%)	19 (21.8%)	
**Comorbid condition, n (%)**						
Hypertension	789 (76.8%)	374 (75.4%)	121 (87.7%)	226 (73.9%)	68 (78.2%)	0.01
Hyperlipidemia	563 (54.8%)	302 (60.9%)	75 (54.3%)	141 (46.1%)	45 (51.7%)	<0.01
Diabetes	312 (30.4%)	142 (28.6%)	49 (35.5%)	94 (30.7%)	27 (31.0%)	0.48
Abdominal pain	214 (20.8%)	100 (20.2%)	31 (22.5%)	70 (22.9%)	13 (14.9%)	0.40
Urologic disease^d^	181 (17.6%)	97 (19.6%)	20 (14.5%)	50 (16.3%)	14 (16.1%)	0.44
**Antihypertensive agents, n (%)**	818 (79.6%)	400 (80.6%)	117 (84.8%)	232 (75.8%)	69 (79.3%)	0.15
ACEi/ARBs	449 (43.7%)	219 (44.2%)	62 (44.9%)	123 (40.2%)	45 (51.7%)	0.27
Beta blockers	427 (41.6%)	212 (42.7%)	62 (44.9%)	118 (38.6%)	35 (40.2%)	0.55
Calcium channel blockers	527 (51.3%)	239 (48.2%)	92 (66.7%)	148 (48.4%)	48 (55.2%)	<0.01

Abbreviations: ACEi, angiotensin-converting enzyme inhibitor; ADPKD, autosomal dominant polycystic kidney disease; ARB, angiotensin receptor blocker; BMI, body mass index; KPSC, Kaiser Permanente Southern California; SD, standard deviation.

a Data shown are n (percentage) or mean (standard deviation).

b *P* values were based on analysis of variance F test for continuous variables and χ^2^ test for categorical variables

c Based on 2015-2019 ACS (American Community Survey) data

d Urologic disease was defined as neoplasm of the bladder, prostate, or ureter, kidney or urinary calculi, urethritis, cystitis, urinary tract infection, and/or urinary retention.

e College degree and household income reflect the area of residence and the respective information from where those patients resided

The mean age at the onset of kidney failure was 55 years and 54.3% were males. Hispanic patients had the youngest age of kidney failure onset at 50 years followed by Asian (53 years), Black (56 years) and White patients (57 years). The proportion of males in each race/ethnicity category was similar with the exception of Asian patients who were more likely to be male (60.9%). Among patients with ADPKD and kidney failure, the overall mean comorbid condition score was 4.7 (2.8). The most common comorbid conditions were hypertension (76.8%), hyperlipidemia (54.8%), and diabetes (30.4%). However, Black patients were most likely to have hypertension (87.7%) and/or diabetes (35.5%) compared to other racial/ethnic groups. The most dispensed medications included calcium channel blockers (51.3%) followed by angiotensin-converting enzyme inhibitors/angiotensin receptor blockers (43.7%) and beta blockers (41.6%) with Black patients (84.8%) having the highest utilization of antihypertensive agents.

**Figure 2:**
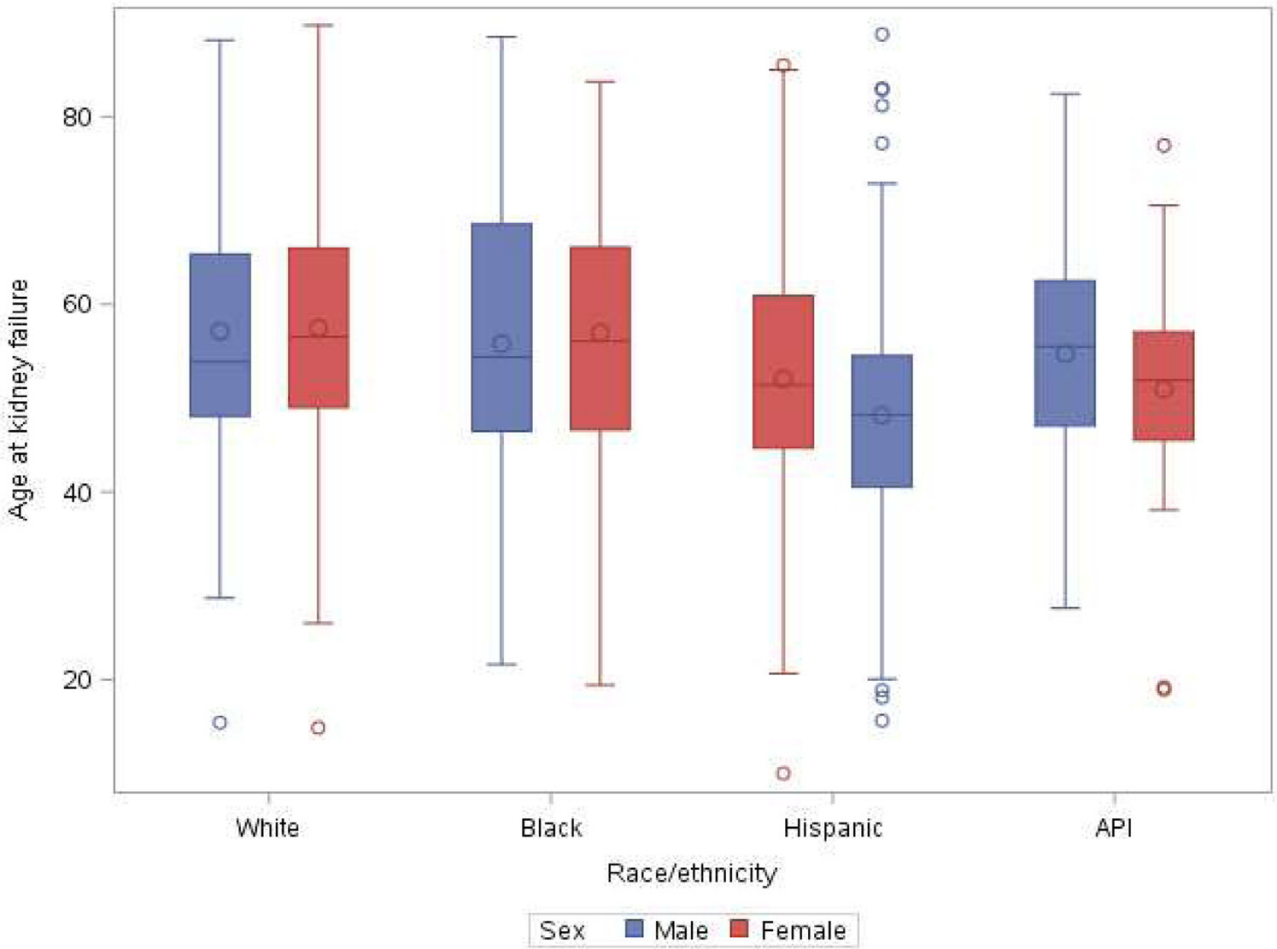
Average age of patients with ADPKD and kidney failure by race/ethnicity and sex. Age of kidney failure onset stratified by race/ethnicity and sex. White, Black, and Hispanic male patients experienced kidney failure at younger ages compared to females: ages 57 vs 58 for White; 56 vs 57 for Black, and 48 vs 52 years for Hispanic patients. Conversely, Asian females had earlier onset of kidney failure compared to males (51 vs 54 years). ADPKD, autosomal dominant polycystic kidney disease.

Age of kidney failure onset stratified by race/ethnicity and sex demonstrated that White, Black, and Hispanic male patients experienced kidney failure at younger ages compared to females: ages 57 vs 58 for White; 56 vs 57 for Black, and 48 vs 52 years for Hispanic patients (Fig 2). Conversely, Asian female patients had earlier onset of kidney failure compared to males (51 vs 54 years).

Among both prevalent and incident kidney failure patients, the mean (standard deviation [SD]) eGFR was 13.4 (15.3) mL/min/1.73 m^2^ with Black patients having the lowest mean (SD) eGFR 10.9 (11.6) mL/min/1.73 m^2^ (Table 2). Black patients had the lowest mean blood laboratory values for hemoglobin (10.8 [1.9] g/dL) and iron saturation 24.1% (8.9) and the highest median parathyroid hormone values (269.5 [152.0-428.0] pg/mL). Bicarbonate values were highest among White and Black patients whereas phosphorus and proteinuria were highest among Hispanic and Asian patients. Albumin and potassium values were similar across race/ethnicity. In a sensitivity analysis only including laboratory values immediately before a kidney transplant or starting dialysis, the mean (SD) eGFR was 8.8 (3.9) mL/min/1.73 m^2^; however, Hispanic and Asian patients had the lowest mean (SD) eGFR with 8.3 (3.3) and 8.3 (3.7) mL/min/1.73 m^2^, respectively (Table S1). Mean blood laboratory values were also lowest among Black patients for hemoglobin and iron saturation, 10.3 (1.7) g/dL and 24.6% (8.9), respectively.

**Table 2:** Laboratory Results for Patients with ADPKD and Kidney Failure by Race and Ethnicity (N = 1,027)

Laboratory^a^	Total	White	Black	Hispanic	Asian/Pacific Islander	*P*^b^
**n (%)**	1,027	496 (47.6%)	138 (13.3%)	306 (29.4%)	87 (8.4%)	
eGFR, mL/min/1.73 m^2^						0.02
n	906	436	129	263	78	
Mean (SD)	13.4 (15.3)	15.0 (16.4)	10.9 (11.6)	12.2 (14.4)	12.5 (16.4)	
Median (Q1, Q3)	8.5 (6.2, 11.9)	9.1 (6.6, 13.6)	8 (6.1, 10.3)	8.3 (6.0, 11.0)	7.9 (5.7, 10.6)	
Hemoglobin, g/dL						<0.01
n	915	438	131	267	79	
Mean (SD)	11.4 (1.9)	11.7 (1.9)	10.8 (1.9)	11.2 (2.0)	10.9 (1.7)	
Median (Q1, Q3)	11.3 (10.2, 12.4)	11.7 (10.5, 12.9)	10.8 (9.8, 12.1)	11 (10.1, 12.3)	11.1 (9.8, 11.9)	
Iron saturation, %						0.39
n	619	286	91	182	60	
Mean (SD)	25.4 (11.4)	25.7 (11.1)	24.1 (8.9)	25.0 (12.9)	27.1 (10.3)	
Median (Q1, Q3)	24.0 (18.0, 31.0)	24 (19.0, 32.0)	24 (19.0, 29.0)	23 (16.0, 31.0)	27 (20.0, 32.0)	
Ferritin, ng/mL						<0.01
n	566	258	86	168	54	
Mean (SD)	314.8 (323.29)	291.8 (279.8)	405.5 (418.5)	265.3 (255.2)	434.4 (458.7)	
Median (Q1, Q3)	200.9 (103.3, 411.0)	201.5 (107.4, 389.0)	253 (117.9, 621.9)	168 (90.7, 375.3)	260.5 (113.7, 613.1)	
Potassium, mEq/L						0.22
n	912	437	130	266	79	
Mean (SD)	4.4 (0.7)	4.5 (0.6)	4.5 (0.7)	4.4 (0.7)	4.4 (0.7)	
Median (Q1, Q3)	4.4 (4.0, 4.9)	4.5 (4.1, 4.9)	4.4 (3.9, 5.0)	4.3 (4.0, 4.7)	4.3 (3.9, 4.9)	
HCO_2_, mEq/L						0.01
n	910	436	130	265	79	
Mean (SD)	22.2 (4.6)	22.5 (4.5)	22.8 (4.8)	21.5 (4.5)	21.5 (4.4)	
Median (Q1, Q3)	22.0 (19.0, 25.0)	22 (19.7, 25.0)	22.5 (19.5, 26.0)	21 (18.0, 24.9)	21.8 (19.0, 24.4)	
Albumin, g/dL						0.42
n	782	387	105	220	70	
Mean (SD)	3.7 (0.6)	3.7 (0.5)	3.6 (0.5)	3.7 (0.6)	3.7 (0.6)	
Median (Q1, Q3)	3.8 (3.4, 4.0)	3.8 (3.4, 4.1)	3.7 (3.2, 4.0)	3.8 (3.4, 4.1)	3.7 (3.4, 4.0)	
Calcium, mg/dL						<0.01
n	873	425	122	251	75	
Mean (SD)	9.0 (0.9)	9.2 (0.8)	9.0 (1.1)	8.8 (1.0)	8.9 (0.9)	
Median (Q1, Q3)	9.1 (8.6, 9.6)	9.2 (8.8, 9.7)	9.1 (8.5, 9.6)	8.9 (8.3, 9.4)	8.9 (8.5, 9.5)	
Phosphorus, mg/dL						0.03
n	837	411	111	244	71	
Mean (SD)	5.2 (1.7)	5.1 (1.7)	5.0 (1.5)	5.4 (1.6)	5.5 (1.9)	
Median (Q1, Q3)	5.0 (4.2, 6.0)	4.9 (4.0, 5.9)	4.8 (4.2, 5.8)	5.3 (4.5, 6.2)	5.2 (4.3, 6.3)	
PTH, pg/mL						<0.01
n	654	311	86	196	61	
Mean (SD)	289.3 (263.2)	237.7 (226.9)	392.7 (369.1)	320.1 (248.9)	308.1 (247.2)	
Median (Q1, Q3)	218.0 (125.0, 362.0)	167 (96.0, 289.0)	269.5 (152.0, 428.0)	257.5 (166.5, 393.5)	248 (151.0, 401.0)	
HbA_1c_, %						0.28
N	422	192	54	137	39	
Mean (SD)	5.9 (0.9)	5.8 (0.9)	5.7 (0.8)	6.0 (1.0)	5.9 (0.7)	
Median (Q1, Q3)	5.6 (5.3, 6.2)	5.6 (5.3, 6.1)	5.6 (5.2, 6.0)	5.7 (5.3, 6.3)	5.8 (5.4, 6.3)	
ALT, U/L						0.03
N	747	365	99	216	67	
Mean (SD)	18.6 (24.8)	17.3 (9.9)	17.2 (8.9)	18.8 (22.9)	26.9 (67.3)	
Median (Q1, Q3)	15.0 (11.0, 20.0)	15 (12.0, 20.0)	16 (12.0, 20.0)	14.5 (11.0, 20.0)	16 (13.0, 22.0)	
AST, U/L						0.79
N	525	250	69	152	54	
Mean (SD)	20.3 (13.3)	20.0 (13.3)	21.1 (7.9)	20.1 (14.5)	21.7 (15.6)	
Median (Q1, Q3)	18.0 (14.0, 22.0)	17.5 (15.0, 21.0)	19 (16.0, 22.0)	17 (13.0, 22.0)	18 (15.0, 23.0)	
Proteinuria^c^, n (%)						0.38
Missing	356 (34.7)	182 (36.7)	50 (36.2)	98 (32.0)	26 (29.9)	
No	58 (5.6)	32 (6.5)	8 (5.8)	16 (5.2)	2 (2.3)	
Yes	613 (59.7)	282 (56.9)	80 (58.0)	192 (62.7)	59 (67.8)	
WBC, n (%)						0.03
Missing	485 (47.2)	242 (48.8)	78 (56.5)	135 (44.1)	30 (34.5)	
No	201 (19.6)	90 (18.1)	27 (19.6)	63 (20.6)	21 (24.1)	
Yes	341 (33.2)	164 (33.1)	33 (23.9)	108 (35.3)	36 (41.4)	
RBC, n (%)						0.01
Missing	499 (48.6)	250 (50.4)	79 (57.2)	140 (45.8)	30 (34.5)	
No	290 (28.2)	127 (25.6)	39 (28.3)	93 (30.4)	31 (35.6)	
Yes	238 (23.2)	119 (24.0)	20 (14.5)	73 (23.9)	26 (29.9)	

*Note:* Patients with kidney failure before January 1, 2002 were excluded from the baseline laboratory results because dialysis or a kidney transplant may have affected their results following treatment.

Abbreviations: ADPKD, autosomal dominant polycystic kidney disease; ALT, alanine aminotransferase; AST, aspartate aminotransferase; RBC, red blood cell; Q, quartile; SD, standard deviation; WBC, white blood cell.

a Laboratory results where available and may not reflect measurements prior to kidney failure. Table S1 reports only laboratory values prior to kidney failure.

b *P* values were based on ANOVA F test for continuous variables, and Fisher's exact test for categorical variables

c Proteinuria was defined as defined as positive for protein in urinalysis (qualitative tests), urine protein quantitation >=200, urine protein/creatinine ratio >0.2 or urine microalbumin/creatine ratio >30.

Overall, 51.2% of patients with ADPKD and kidney failure had a kidney transplant (Table 3). Across race/ethnic groups, more White (53.8%) and Asian (52.9%) patients had a kidney transplant compared to any other race/ethnicity. Black (44.2%) and Hispanic (49.7%) patients had the lowest proportion of kidney transplants. Of all of the patients with ADPKD with kidney failure, 15.0% had a preemptive kidney transplant. Across race/ethnicity, 19.0% of White, 16.1% of Asian, 13.4% of Hispanic, and 3.6% of Black patients underwent a preemptive kidney transplant. Multivariable logistic regressions analyses demonstrated that compared to White patients, the ORs for having a kidney transplant were 0.72 (0.45-1.12) and 0.65 (0.45-0.94) for Black and Hispanic patients, respectively (Table 4). Older age and higher Elixhauser score were also associated with lower transplant OR.

**Table 3:** Kidney Transplant Among Patients with ADPKD and Kidney Failure

Characteristics	Total	White	Black	Hispanic	Asian/Pacific Islander	*P*^a^
**N (%)**	1,027 (100.0)	496 (48.3)	138 (13.4)	306 (29.8)	87 (8.5)	
**Kidney transplant**^b^**, n (%)**	526 (51.2)	267 (53.8)	61 (44.2)	152 (49.7)	46 (52.9)	0.22
**Kidney transplant at kidney failure onset/preemptive transplant**	154 (15.0)	94 (19.0)	5 (3.6)	41 (13.4)	14 (16.1)	<0.01

Abbreviation: ADPKD, autosomal dominant polycystic kidney disease.

a *P* values were based on χ^2^ test

b Dialysis may precede transplant.

**Table 4:** Factors Associated with Kidney Transplant Among Patients with ADPKD and Kidney Failure

	Odds Ratio (95% Confidence Interval)^a^	
	Kidney Transplant (*Dialysis May Precede Transplant)*	Kidney Transplant at Kidney Failure Onset (Preemptive Transplant)
**Race/ethnicity**		
White	Reference	Reference
Asian/Pacific Islander	0.79 (0.46, 1.35)	0.70 (0.36, 1.38)
Black	0.72 (0.45, 1.12)	0.17 (0.07, 0.44)
Hispanic	0.65 (0.45, 0.94)	0.61 (0.38, 0.99)
**Sex**		
Male	Reference	Reference
Female	1.17 (0.88, 1.57)	1.25 (0.85, 1.83)
**Age at kidney failure**^b^	0.95 (0.93, 0.96)	0.97 (0.95, 0.99)
**BMI at kidney failure**		
Normal weight (BMI < 25)	Reference	Reference
Overweight (BMI 25-30)	1.45 (0.91, 2.32)	1.61 (0.77, 3.33)
Obesity (BMI ≥ 30)	0.75 (0.45, 1.25)	1.10 (0.46, 2.61)
Unknown	1.91 (1.27, 2.87)	1.51 (0.8, 2.84)
**Elixhauser score**	0.80 (0.75, 0.85)	0.71 (0.65, 0.79)
**Census-level college degree**^c^		
<50%	Reference	Reference
50-75%	1.32 (0.91, 1.91)	1.15 (0.67, 2.0)
>75%	1.69 (1.04, 2.73)	1.54 (0.81, 2.95)
Unknown	0.72 (0.23, 2.32)	2.70 (0.56, 13.13)
**Census-level median household income**^c^		
<$45,000	Reference	Reference
$45,001-$80,000	1.05 (0.63, 1.74)	1.57 (0.65, 3.78)
>$80,000	1.13 (0.66, 1.95)	2.23 (0.9, 5.53)
Unknown	1.20 (0.50, 2.88)	1.12 (0.27, 4.60)

Abbreviations: ADPKD, autosomal dominant polycystic kidney disease; BMI, body mass index.

a Adjusted for age, sex, BMI, Elixhauser Comorbidity Index and Census-level education and income

b Age is continuous variable and reflect per every 1 year increasing age

c College degree and household income reflect the area of residence and the respective information from where those patients resided

## Discussion

Our study describes one of the most diverse ADPKD cohorts to date and includes both Hispanic and Asian patients. We observed racial/ethnic differences among patients with ADPKD and kidney failure where 27.3% of prevalent ADPKD patients had kidney failure. The mean age at kidney failure onset was 54.6 years. Male patients experienced kidney failure at younger ages compared to females. Hispanic and Asian patients with ADPKD had a lower prevalence of kidney failure but had earlier kidney failure onset compared to White and Black patients. The majority (51.2%) of our kidney failure ADPKD population had undergone kidney transplantation, but transplant rates varied across race/ethnicities as well.

The mean eGFR of the ADPKD cohort before kidney failure was 8.8 mL/min/1.73 m^2^. This was lower than the mean eGFR of 9.4 mL/min/1.73 m^2^ for KPSC patients with kidney failure from all causes.^25^ Lab findings were also notable for our ADPKD cohort in that the mean albumin was higher (3.7 g/dL) compared to all KPSC incident kidney failure patients (3.1 g/dL). Even when compared to KPSC kidney failure patients in the age group 45-64 years (3.2 g/dL). These findings are consistent with the fact that ADPKD patients are generally younger and healthier versus other kidney failure populations before starting kidney replacement therapy.

Our study findings highlight a potential role of race/ethnicity in the age of onset and progression of ADPKD. The prevalence of kidney failure was lower in Asian and Hispanic compared to White and Black patients. However, Asian and Hispanic patients with ADPKD were younger at kidney failure onset. One possible explanation may be a variability in genetic penetrance and interplay across different race/ethnicities. Modifier genes have been theorized to play a role within-family variations in ADPKD clinical manifestations including earlier onset of kidney failure.^26^, ^27^, ^28^ Prior studies have described mutations associated with PKD1 such as the truncating protein mutations affecting the progression of kidney failure.^29^ Both location and type of mutation within PKD1 were demonstrated to influence kidney survival.^11^ Race/ethnicity may represent a modifier in terms of its association with the penetration of ADPKD genes. Currently, the Retrospective epidemiological study of Asia-Pacific patients with rapId Disease progression of Autosomal Dominant Polycystic Kidney Disease (RAPID-ADPKD) is underway in the Asia-Pacific region to identify epidemiological data on clinical manifestations and disease progression in ADPKD populations in this region.^30^

Race/ethnicity may serve as a marker for socioeconomic, behavioral, and genetic risk factors that have the potential to impact ADPKD disease progression and outcomes. Patient environment and health behaviors may also have contributed to variation in the timing of kidney failure onset. Cultural differences that affect lifestyle, diet, and preventive health care practices may be related to some of the differences observed. In general, higher poverty rates and lower socioeconomic status have been associated with progression of chronic kidney disease (CKD).^31^ Despite the reduction in the financial barriers and other factors that affect access to care in the advent of Affordable Care Act, disparities in health care between racial/ethnic groups continue to exist.^32^ One surrogate of health care that we evaluated was the mean length of KPSC membership in our ADPKD cohort with kidney failure. The shorter length of membership among Hispanic and Asian ADPKD patients (Table 1) may reflect shorter duration of CKD care provided to these patients, potentially contributing to earlier onset kidney failure.

The higher kidney failure prevalence seen in Black patients may also be attributable to higher rates of comorbid conditions such as hypertension, diabetes, and other genetic conditions.^33^ In our study population, the prevalence of hypertension and diabetes was highest among Black patients. Although we did not have information on *Apolipoprotein L1* (*APOL1*) risk gene mutation status, the mutation is present in approximately 13% of African Americans in the United States and may have contributed to higher rates of kidney failure in our Black population.^34^^,^^35^

In our ADPKD kidney failure population, 51.2% had undergone kidney transplant. Compared to other patients with CKD, patients with ADPKD are generally recognized earlier and subsequently undergo management by nephrologists earlier. Thus, timely and perhaps more lengthy CKD care may lead to more comprehensive transplant education and awareness of ADPKD. In addition, patients with ADPKD often have isolated kidney disease, making them more transplant eligible compared to the general kidney failure population, who carry high morbidity burdens. All of these factors could result in earlier waitlisting of qualified candidates for kidney transplantation especially within an insured population.^9^

Racial/ethnic disparities among the ADPKD population were observed for transplantation as well. White and Asian patients had the highest rate of kidney transplant at 53.8% and 52.9%, respectively. Black patients had the lowest rate of kidney transplant at 44.2%. Among our study cohort, 15.0% underwent preemptive kidney transplant, which was a much higher rate in comparison to the rate of 2.9% in the United States for all-cause kidney failure patients.^5^ Preemptive kidney transplants were highest among White patients (19.0%) and lowest among Black patients (3.6%). Overall, Black and Hispanic ADPKD patients had 28%-35% lower likelihood of transplant. Although not a direct comparison, analyses from the United States Renal Data System (USRDS) also reported differences in transplant for all-cause kidney failure patients where Black and Hispanic patients had lower rates of transplant compared to Whites and Asian patients.^5^^,^^20^ McGill et al^20^ also reported lower transplant rates in Black and Hispanic patients compared to White patients. They observed that ADPKD patients with private insurance were 2-3 times more likely to receive a transplant.^20^ All patients in our study had insurance coverage, but nevertheless, the likelihood of transplant was still lower among Black and Hispanic compared to White patients, although the ORs were not as low as the USRDS observation. Thus, having insurance appears to attenuate some disparities in transplantation, but race/ethnic disparities seem to persist.

Our study has several limitations that may affect the interpretation of our findings. First, we used diagnosis codes to identify ADPKD; therefore, some patients may have been misclassified, which could result in over- or underreporting ADPKD prevalence. However, the computable phenotype has been demonstrated to have a high level of sensitivity and specificity.^36^ Second, we did not have information on abdominal imaging with age-adjusted total kidney volume for our study population. Third, categories of race/ethnicity do not accurately reflect underlying genetic or biological distinctions in populations.^37^ The American Medical Association recently adopted a policy recognizing race as a social construct and recommending clinicians and researchers to consider genetics and biology, potential structural racism and racialized medical practices, and social determinants of health when describing risk factors.^38^ In our study, we did not have genetic information available for our entire population, which would provide important diagnostic and prognostic information in ADPKD. Key drivers of health disparities that are often inadequately addressed include neighborhoods, housing, environmental exposures, education, and access to quality care, medications, and healthy food options.^39^^,^^40^ Racialized medical practices such as the incorporation of race in clinical risk equations may have exacerbated health disparities seen in kidney disease and other medical conditions. There has been longstanding inclusion of a “race correction coefficient” in numerous equations estimating kidney function via eGFR, specifically for African Americans.^41^ Recently, it was determined that this “race correction coefficient” could lead to overestimation of kidney function in African Americans thereby delaying diagnosis and management of CKD.^42^ In our study, kidney function calculations were based on equations incorporating race. Another limitation is that patients may have disenrolled from KPSC shortly before initiating kidney transplant, which could have underestimated the proportion of patients with ADPKD and kidney failure who underwent kidney transplant. Finally, our study was conducted within a single, integrated health care system in one geographic region and may not be generalizable to all patients with ADPKD.

Despite these potential limitations, our study is one of the first to date to include Hispanic and Asian patients with ADPKD. Our findings captured within a real-world clinical environment may be more representative than findings from clinical trials or prospectively enrolled population studies. The electronic health record at KPSC is comprehensive where it captures laboratory information, medications, and health care utilizations of all members.

In conclusion, among a large diverse ADPKD population from the southwestern United States, we observed racial/ethnic differences in rates of kidney failure, age of kidney failure onset, and rates of kidney transplantation. Overall, Hispanic patients had the lowest kidney failure rates but had the youngest age at kidney failure onset. Although overall kidney transplants and preemptive transplant rates were high in this ADPKD population, Black patients had the lowest rates of both. Our study cohort from a real-world setting provides insight into identifying high risk ADPKD patients and raises many questions for future research on the role of race in ADPKD progression, as well as potential disparities in care. More insights into understanding ADPKD progression to kidney failure among different races and ethnicities may lead to improved and individualized management strategies for this CKD population.

## Footnotes

**Supplementary File (PDF)**
**Table S1:** Laboratory Results in the Year before Kidney Failure for Patients with ADPKD and Kidney Failure, by Race and Ethnicity
