# Supplementary material for: Health Disparities in Kidney Failure Among Patients With Autosomal Dominant Polycystic Kidney Disease: A Cross-Sectional Study
Source: Kidney Med. 2022 Dec 5;5(2):100577. doi: 10.1016/j.xkme.2022.100577 (PMC9883284; doi:10.1016/j.xkme.2022.100577)
Supplement: Supplementary File (PDF) — Table S1. [file mmc1.pdf]

Table S1. Laboratory Results in the Year Prior to Kidney Failure for Patients with ADPKD and Kidney Failure, by Race and Ethnicity (N=736)

Laboratory†	Total (N=736)	White (N=345)	Black (N=95)	Hispanic (N=230)	Asian/Pacific Islander (N=66)	P-Value‡
eGFR, mL/min/1.73m^2						0.02
N	672	316	89	206	61	
Mean (SD)	8.8 (3.9)	9.3 (4.4)	8.5 (3.4)	8.3 (3.3)	8.3 (3.7)	
Median (Q1, Q3)	8.5 (6.3, 10.6)	8.7 (6.6, 11.4)	8.3 (6.2, 10.2)	8.3 (6.0, 10.5)	7.9 (5.9, 10.2)	
Hemoglobin, g/dL						<0.01
N	672	316	89	206	61	
Mean (SD)	10.9 (1.7)	11.2 (1.7)	10.3 (1.7)	10.7 (1.7)	10.6 (1.6)	
Median (Q1, Q3)	10.9 (9.9, 11.9)	11.3 (10.2, 12.2)	10.4 (9.4, 11.3)	10.7 (9.9, 11.8)	10.8 (9.7, 11.6)	
Iron Saturation, %						0.63
N	530	244	73	161	52	
Mean (SD)	25.6 (11.5)	25.8 (11.2)	24.6 (8.9)	25.4 (13.2)	27.2 (10.9)	
Median (Q1, Q3)	24.0 (18.0, 31.0)	24 (19.0, 32.0)	24 (19.0, 29.0)	23 (16.0, 31.0)	27.5 (20.0, 32.5)	
Ferritin, ng/mL						0.01
N	486	222	69	149	46	
Mean (SD)	278.3 (261.8)	273.9 (244.3)	317.5 (290.5)	237.6 (221.7)	372.5 (373.0)	
Median (Q1, Q3)	194.0 (100.3, 378.0)	197 (104.0, 384.0)	198.1 (117.9, 416.0)	151.3 (86.9, 349.3)	243.2 (111.4, 524.0)	
Potassium, meq/L						0.01
N	672	316	89	206	61	
Mean (SD)	4.4 (0.6)	4.5 (0.6)	4.5 (0.7)	4.3 (0.6)	4.4 (0.6)	
Median (Q1, Q3)	4.4 (4.0, 4.9)	4.5 (4.1, 4.9)	4.4 (4.1, 5.0)	4.3 (3.9, 4.7)	4.3 (4.0, 4.8)	
HCO2, meq/L						0.11
N	672	316	89	206	61	
Mean (SD)	20.7 (4.0)	21.1 (4.0)	20.8 (3.9)	20.3 (3.9)	20.3 (4.0)	
Median (Q1, Q3)	21.0 (18.0, 23.0)	21 (19.0, 23.0)	21 (19.0, 23.0)	20 (18.0, 23.0)	21 (17.0, 23.0)	
Albumin, g/dL						0.53
N	611	296	79	180	56	
Mean (SD)	3.7 (0.5)	3.7 (0.5)	3.7 (0.5)	3.8 (0.6)	3.7 (0.5)	
Median (Q1, Q3)	3.8 (3.4, 4.1)	3.8 (3.4, 4.1)	3.8 (3.3, 4.0)	3.9 (3.6, 4.1)	3.7 (3.4, 4.0)	
Calcium, mg/dL						<0.01

N	663	315	87	201	60	
Mean (SD)	8.9 (0.9)	9.0 (0.8)	8.9 (1.1)	8.7 (1.0)	8.7 (0.8)	
Median (Q1, Q3)	9.0 (8.5, 9.4)	9.1 (8.6, 9.5)	9 (8.4, 9.4)	8.8 (8.3, 9.3)	8.8 (8.4, 9.3)	
Phosphorus, mg/dL						0.30
N	646	308	83	197	58	
Mean (SD)	5.5 (1.5)	5.4 (1.7)	5.2 (1.3)	5.6 (1.4)	5.6 (1.7)	
Median (Q1, Q3)	5.2 (4.5, 6.1)	5.1 (4.4, 6.1)	4.9 (4.4, 5.9)	5.4 (4.7, 6.2)	5.3 (4.4, 6.3)	
PTH, pg/mL						<0.01
N	577	277	70	177	53	
Mean (SD)	268.3 (221.1)	221.8 (189.5)	341.2 (314.7)	307.8 (217.7)	283.7 (186.5)	
Median (Q1, Q3)	210.0 (125.0, 343.0)	167 (98.0, 274.0)	258 (151.0, 392.0)	247 (169.0, 392.0)	236 (145.0, 401.0)	
A1c, %						0.41
N	355	163	42	115	35	
Mean (SD)	5.8 (0.8)	5.7 (0.8)	5.8 (0.9)	5.9 (0.9)	5.9 (0.6)	
Median (Q1, Q3)	5.6 (5.3, 6.1)	5.5 (5.3, 5.9)	5.7 (5.2, 6.2)	5.6 (5.3, 6.2)	5.8 (5.4, 6.3)	
ALT, U/L						0.02
N	563	266	71	172	54	
Mean (SD)	17.8 (27.4)	16.0 (8.6)	17.0 (9.5)	17.4 (23.1)	29.0 (74.7)	
Median (Q1, Q3)	14.0 (11.0, 19.0)	14 (11.0, 19.0)	16 (12.0, 19.0)	14 (11.0, 18.5)	16.3 (13.0, 22.0)	
AST, U/L						0.27
N	365	171	43	110	41	
Mean (SD)	19.4 (12.3)	18.6 (12.1)	21.1 (9.1)	18.8 (11.3)	22.2 (17.5)	
Median (Q1, Q3)	17.0 (13.0, 21.0)	16 (13.0, 20.0)	19 (15.0, 22.0)	16 (13.0, 21.0)	18 (15.0, 23.0)	
Proteinuria*, n (%)						0.75
Missing	106 (14.4)	52 (15.1)	15 (15.8)	33 (14.3)	6 (9.1)	
No	44 (6.0)	21 (6.1)	7 (7.4)	14 (6.1)	2 (3.0)	
Yes	586 (79.6)	272 (78.8)	73 (76.8)	183 (79.6)	58 (87.9)	
WBC, n (%)						0.08
Missing	213 (28.9)	105 (30.4)	35 (36.8)	63 (27.4)	10 (15.2)	
No	194 (26.4)	85 (24.6)	27 (28.4)	62 (27.0)	20 (30.3)	
Yes	329 (44.7)	155 (44.9)	33 (34.7)	105 (45.7)	36 (54.5)	
RBC, n (%)						0.02
Missing	227 (30.8)	113 (32.8)	36 (37.9)	68 (29.6)	10 (15.2)	

No	279 (37.9)	118 (34.2)	39 (41.1)	91 (39.6)	31 (47.0)
Yes	230 (31.3)	114 (33.0)	20 (21.1)	71 (30.9)	25 (37.9)

† Data shown are N (percentage) or mean (standard deviation).
* Proteinuria was defined as defined as positive for protein in urinalysis (qualitative tests), urine protein quantitation >=200, urine protein/creatinine ratio >0.2 or urine microalbumin/creatine ratio >30.
‡P-Values were based on ANOVA F-test for continuous variables, and Fisher's exact test for categorical variables
